# Childcare practices among teenage mothers in Ghana: a qualitative study using the ecological systems theory

**DOI:** 10.1186/s12889-020-09889-7

**Published:** 2021-01-04

**Authors:** Reuben Foster Twintoh, Prince Justin Anku, Hubert Amu, Eugene Kofour Maafo Darteh, Kwaku Kissah Korsah

**Affiliations:** 1 Department of Population and Health, College of Humanities and Legal Studies, University of Cape Coast, Cape Coast, Ghana; 2 Department of Population and Behavioural Sciences, School of Public Health, University of Health and Allied Sciences, Hohoe, Ghana

**Keywords:** Childcare practices, Lived experiences, Teenage mothers, Ecological systems theory (EST), Ghana

## Abstract

**Background:**

While appropriate care for children is essential for optimal growth and protection against child morbidity and mortality, teenage mothers have been shown to deviate from the recommended childcare practices. This study explored the childcare practices among teenage mothers in Ghana using Ecological Systems Theory by Bronfenbrenner as a theoretical framework.

**Methods:**

Employing qualitative approach to inquiry, evidence was drawn from 30 teenage mothers using in-depth interviews. The data were analysed and presented following systematic qualitative-oriented text analysis strategy with verbatim quotes from study participants to support the emergent themes.

**Results:**

It was evident that teenage mothers have limited skills in childcare practices and often resorted to practices with potentially adverse health outcomes for their children. They, for instance, applied hot towels they had heated with hot stones to the children’s umbilical stump. We found that teenage mothers were not in sync with their macro- and exo-systems, thereby depriving themselves and their babies of the much-needed guidance and support in caring for their babies. Teenage mothers were often confused and sometimes clueless about best childcare practices at a given point in time.

**Conclusions:**

Childcare practices by teenage mothers are far from the ideal. To improve on child health (especially children born to teenage mothers), efforts at both the macro- and exo-systems should be directed at exposing teenage mothers to best child care practices that inure to the benefits of their children. Ante- and postnatal visits should be used to provide specific education for mothers, especially first-time teenage mothers on the care needs of babies and how to provide these needs.

**Supplementary Information:**

The online version contains supplementary material available at 10.1186/s12889-020-09889-7.

## Background

Globally, about 5.3 million under-five children died in 2018 [1]. More than half of these deaths were preventable and these children could have survived by simple, accessible and affordable interventions including adequate nutrition and appropriate childcare practices such as exclusive breastfeeding, recommended complementary feeding, child bathing and cleaning, proper sanitation and hygiene practice [1, 2]. However, teenage mothers have been found to deviate from the standards and recommended infants and childcare practices [1, 3, 4].

In Ghana, it has been documented that about 28% of all under-five children are stunted (as a result of chronic malnutrition) and 40% of these cases involved children aged 18 to 23 months [5]. Several incidences of child illnesses, malnutrition, and developmental delays, especially among children born to teenage mothers have been reported in the country [6–8]. The need for improved and sustained effort in halting and reversing the situation is not only critical for ensuring and improving the health of children, but also at the core of achieving the Sustainable Development Goals (SDGs) goal 3, which seeks to ensure healthy lives and promote well-being for all at all ages. Target 3.2 of the goal specifically seeks to end preventable death of new-borns and children under 5 years of age [9]. Also, the new global strategy for women’s, children’s and adolescents’ health (2016 to 2030) agenda seeks to ensure that women and their babies survive and reach their full health potentials in life [10]. Appropriate childcare practices such as exclusive breastfeeding, complementary feeding, child bathing and cleaning, umbilical cord care, and sleeping pattern or arrangement of children are beneficial and therefore recommended for children [4, 11].

In some communities in Ghana, childcare is influenced by culture and some traditional practices that are transmitted from older mothers to the young ones (daughters) throughout the generations [10]. Young mothers required knowledge and assistance to be able to perform their maternal roles and cope with motherhood in light of the challenges they face [12]. These complex dynamics continue to shape how mothers, especially teenage mothers care for their babies. Therefore, an in-depth understanding of teenage mother’s childcare practices within the family structures and cultures is thus worthwhile exploring. Besides, policymakers and health professionals must acquire a deeper understanding of the diverse childcare practices to better appreciate and interact with teenage mothers, bearing in mind the dynamics. Our study is situated within the Ecological System Theory to offer a broader contextual understanding of the childcare practices among teenage mothers.

### Theoretical framework

The Ecological Systems Theory (EST) was adapted as the framework for the study. Propounded in 1979 by Urie Bronfenbrenner, the theory recognises the ecology of human growth and development and draws heavily on complex interplay of individual (teenage mother) relationship, socio-cultural and environmental factors to understand the role of motherhood [13]. According to Bronfenbrenner, an ecological perspective encourages individuals to consider the holistic environment for themselves. These factors must be considered in terms of their existence at each level and their interactions across each system [13]. In this study, teenage mothers were placed at the centre of their social context, and their lived experiences with childcare practices were discussed. The EST identifies the importance of the immediate environment (microsystems) through to the broader layers; exo, macro and chrono systems. The first ecological system, which incorporates the immediate environmental surroundings of the individual and those with whom the individual (teenage mother) interact such as the parent, neighbourhood, peers, and baby’s father focuses on the support and the interaction/relationship that exist among them. The second layer is the mesosystem, which fundamentally underline and incorporate the relationship that occurs within the microsystem. For instance, the interaction between the individual (teenage mother) and health service or education as a support network. This web of relationships is associated with a positive influence on the family or individual (teenage mother) and the baby [14].

**Fig. 1:**
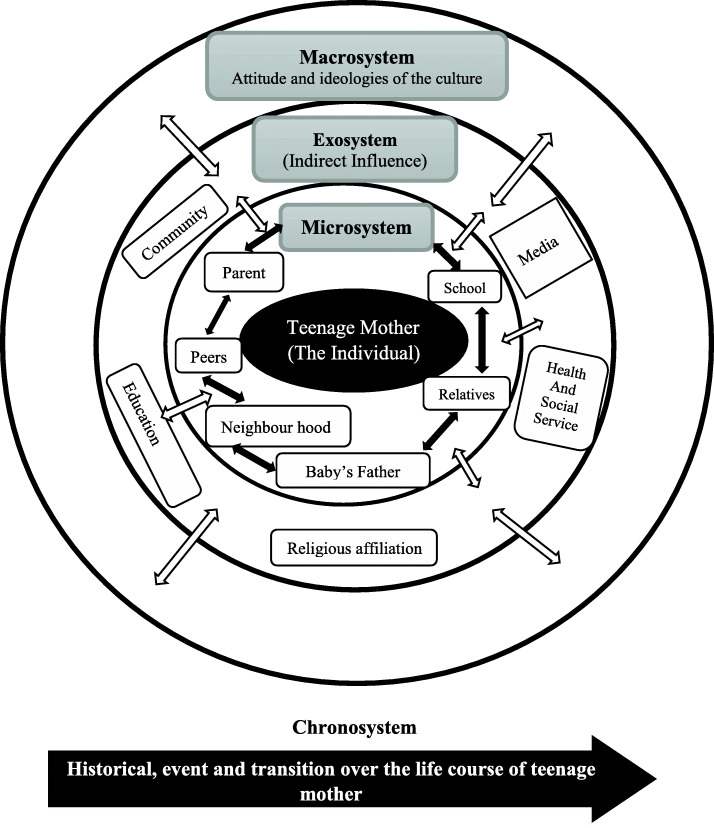
Adapted Ecological Systems Theory (EST). Source: Bronfenbrenner (1979). To the best of our knowledge, Fig. 1 in this adapted form has never been published elsewhere. Therefore, no permission is required for its publication as part of this manuscript

The macrosystem refers to the larger social and cultural environment in which all the other systems exist. It consists of the wider environment and draws heavily on attitude, ideologies, culture and beliefs that have indirect effects on other systems and the individual. Thus, it involves teenage mothers’ child care practices and motherhood, where their closest individuals provide support, skills, and guidance to sustain them in light of the challenges they face regarding motherhood and their effects over the life course transitions of the teenage mothers at the chronosystem. The EST has been modified holistically to help in understanding the domain and the impact of the health and social contexts on childcare practices and experiences of teenage mothers. Here, challenges, constraints, coping strategies, support or a combination of both positive and negative experiences exist. The experiences of being a teenage mother are shaped at many levels. As such, the EST provides a framework to explore these levels and the interactions between and among them. This makes the framework useful as it includes multi-dimensional social context and provides a multi-layered approach for data analysis (see Fig. 1). EST has been widely used to study various social phenomena and its power in offering meaningful insight into social problems has been adequately documented [15–17].

## Methods

### Setting

**Fig. 2:**
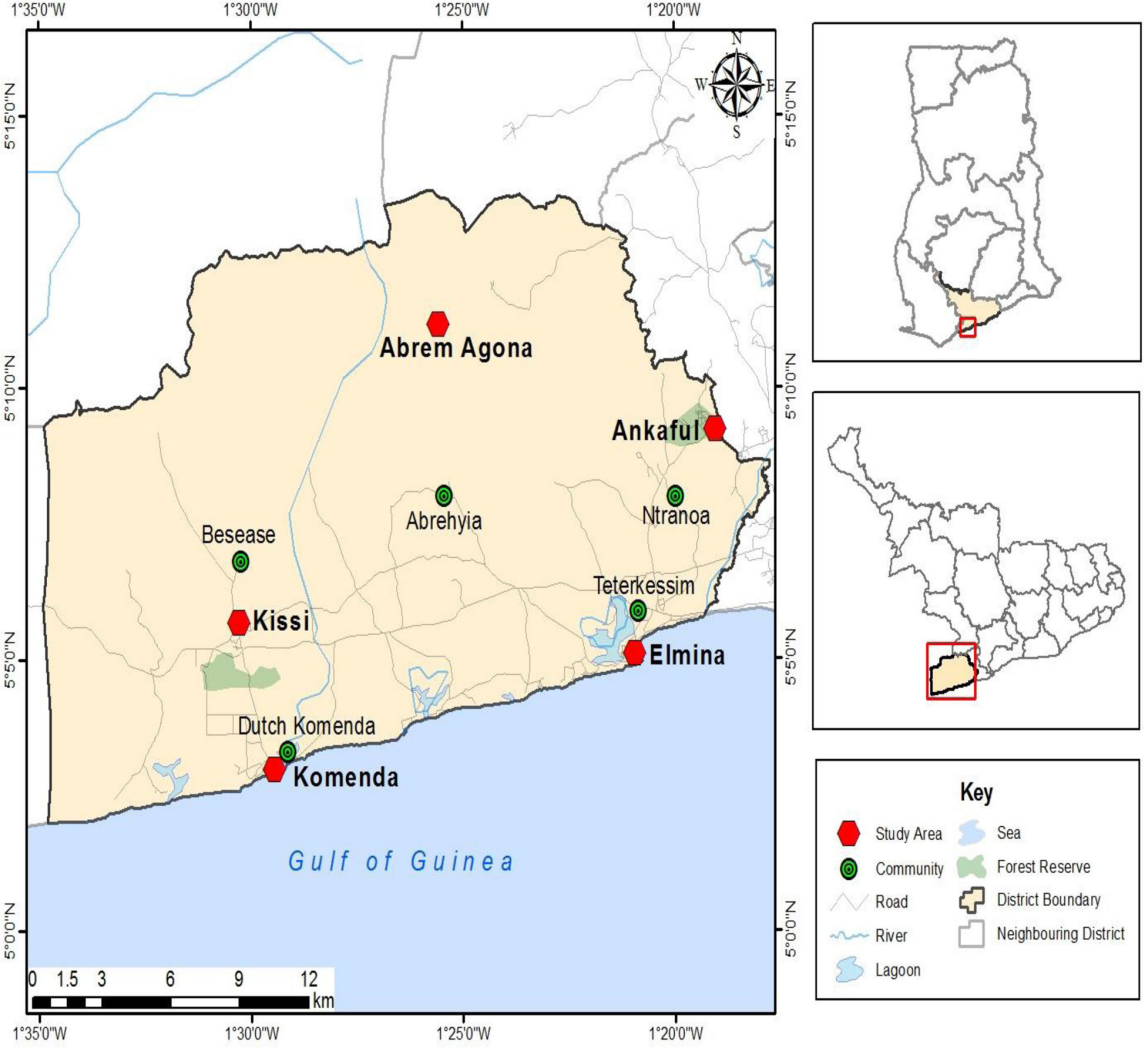
Map of the Study Area. Source: GIS Lab of the Department of Geography and Regional Planning, University of Cape Coast (2019). The map of the study area was specifically developed for this study. Permission has been obtained for it to be published as part of the manuscript

The study was conducted in the Komenda-Edina-Eguafo-Abrem (KEEA) Municipality in the Central Region of Ghana. The municipality is bounded on the south by the Atlantic Ocean (Gulf of Guinea), the east by the Cape Coast Metropolis, the north by the Twifo Hemang - Lower Denkyira District and the west by the Mpohor - Wassa East District and Shama District. Perched between longitude 1^o^ 20′ West and 1^o^ 40′ West and latitude 5^o^ 05′ North and 5^o^ North 15′ North, the municipality covers an area of 1′372.45 km^2^ 919.95 mile^2^ [18]. The KEEA has four traditional states and further divided into 6 zonal councils with 54 electoral areas, 11 sub-communities with Elmina being the municipal capital which became independent in 1988. Fishing, farming and salt winning/making are the main economic development activities. The KEEA municipality has one of the highest incidences of teenage pregnancy in Ghana [19]. Map of the Study Area (Fig. 2).

### Study design

This study employed phenomenology as a study design with its attendant qualitative methods as they provided us with the necessary tools to explore the phenomenon from the perspective of teenage mothers drawing on their lived experiences [20]. Phenomenology is not only concerned with a description of the phenomenon but also an interpretive process. In effect, we connect the different meanings of the lived experiences and perspectives shared by teenage mothers (see [21]). Phenomenology has been widely used across the humanities and social sciences where the design has demonstrated enormous strength in helping to construct meaning into people’s experiences and unique perspectives [22, 23]. With the aid of this design, we were able to obtain appropriate data that offered us a deeper understanding of child care practices from the perspective of our study participants.

### Population

The study targeted teenage mothers aged 13 to 19 years in the KEEA Municipality in the central region with a child less than 2 years at the time of the fieldwork. This was done to safeguard against recall biases as it will be difficult for a mother to recall vividly their daily childcare practice after 2 years. Also, teenage mothers with children more than 2 years might have passed the childcare practices stage, for instance, breastfeeding (exclusive and complementary breastfeeding) and may not be able to contribute in this regard. As such, we aimed at participants that could provide us with in-depth information that covered a wide range of childcare practices.

### Sampling and sample size

Teenage mothers were purposively selected from the five communities in the KEEA Municipality. In all, thirty (30) teenage mothers were recruited for the study. Guided by the concept of saturation, the sample size was not determined a priori but evolved during the data collection. The point of saturation was based on the recommendation by Marshall et al. [24] who demonstrated that theoretical saturation mostly occurs between 10 and 30 interviews.

### Data collection

The research instruments used for this study were in-depth interview guide (IDI) and pictorial diary. The IDI guide and the pictorial diary were specifically developed by the authors for this study. The IDI guide was broadly categorised into two sections. The first section (section 1) looked at the teenage mothers’ socio-demographic characteristics: teenage mothers’ age, level of education, religion, marital status, and current occupation. The second section (section 2) focused on teenage mother’s childcare practices**.** Also, “pictorial-guide interview method”, which combines photos and/or illustrations in support of the in-depth interviews was employed. We first administered the pictorial diary where we took notice of all perspectives shared by the participants and then explored the issues further in the in-depth interviews. Using both the interview guide and the pictorial diary (images) methods augment data collection and ensured completeness (see [25, 26]). Not only did the pictorial guide served the function of supplementing the interviews (although of varying quality), but also provided us with the opportunity to gain at least some modicum of access to naturally occurring events whose meanings were then explored in the in-depth interviews [27].

Before the commencement of the interview, the participants were informed about the purpose of the study. In order to encourage the participants to open up, the interviewer re-iterated that their right to anonymity and confidentiality would be respected. As such, their names will not be attached to the data or included in the final report of the study. The selected participants consented to participate freely. The interviewer read and explained the information sheet and the informed consent form to the participant in the language (Akan/Fante or English) they best understood. Participants were asked to either sign or thumbprint a consent form indicating they have willingly chosen to participate in the study. All interviews were conducted by the first author and supervised by EKMD.

The face-to-face interview method was employed during the data collection in the homes of the participants to ensure that participants feel most comfortable to respond adequately to the questions. The interviews were based on one-on-one interaction between the interviewer and the participant alone. All interviews were tape-recorded and field notes were taken during the interviews. Each interview lasted for about 55 min on the average.

### Data analyses

The data were analysed using systematic qualitative oriented text analysis [28] with the assistance of QSR NVivo 12 Pro software for qualitative data analysis. First, all audio-recorded interviews were transcribed verbatim and field notes were typed into Microsoft Word and then imported into the software to help create parent and child nodes. The issues were described, interpreted, summarized and organized to demonstrate the key issues that were identified from the data for analysis [28, 29]. A numeric scheme of coding was employed to mark all parts of the written discourse that contained one category or another (classification). 1, for instance, was used to code ‘Exclusive breastfeeding’, 2 for ‘Complementary feeding’, 3 for’ Benefits of breastfeeding’ and so on. These categories were then reduced into a much smaller size by grouping similar and related themes to arrive at comprehensive new categories.

The initial coding was independently done by RFT and PJA. Codes were later compared and the few inconsistencies were mutually resolved. Systematic qualitative-oriented text analysis was used to develop a framework for analysis to gain an intuitive sense of the data and to determine our approach to coding the data rather than have it managed solely by the software. Therefore, we made use of the “Query” function of the software to search for specific words and phrases that were critical to the observed themes [see 30]. In the process of analysis, forty-three quotes/codes and nine categories/subthemes were obtained. Specific participant’s quotations were represented by unique identifiers R1 to R30. Quotations from participants were used to support the final emergent themes. All the conceptual expressions that emerged from participants were categorised in a way that reflected the salient and subtle meaning participants attached to their expressions. The consolidated criteria for reporting qualitative research (COREQ, see Additional file 1: appendix A) was adopted in reporting our study results [30].

## Results

### Socio-demographic characteristic of participants

Table 1 represents the socio-demographic characteristics of the teenage mothers. Ten per cent of the study participants have completed Senior High School and were married, approximately 64% of them were never married. Ninety per cent of the participants were Christian. Half of the participants had no occupation while 20% engaged in petty trading with others involved in vocational and mobile banking services. A summary of the background characteristics of the study participants is provided in Table 1.

**Table 1:** Socio-demographic Characteristics of participants

Variable	Frequency (N) (*N* = 30)	Percentage (%)
**Age (In completed years)**		
16	3	10.0
17	6	20.0
18	8	26.7
19	13	43.3
**Level of Education**		
Primary	9	30.0
JHS	18	60.0
SHS	3	10.0
**Marital Status**		
Never married	19	63.3
Married	3	10.0
Cohabiting	8	26.7
**Religion**		
Christian	27	90.0
Islamic	3	10.0
**Occupation**		
Not working	15	50.0
Petty trading	6	20.0
Seamstress	4	13.3
Hairdresser	3	10.0
Mobile banking/Transfer	2	6.7

Source: Fieldwork, 2019

### Childcare practices of teenage mothers

**Fig. 3:**
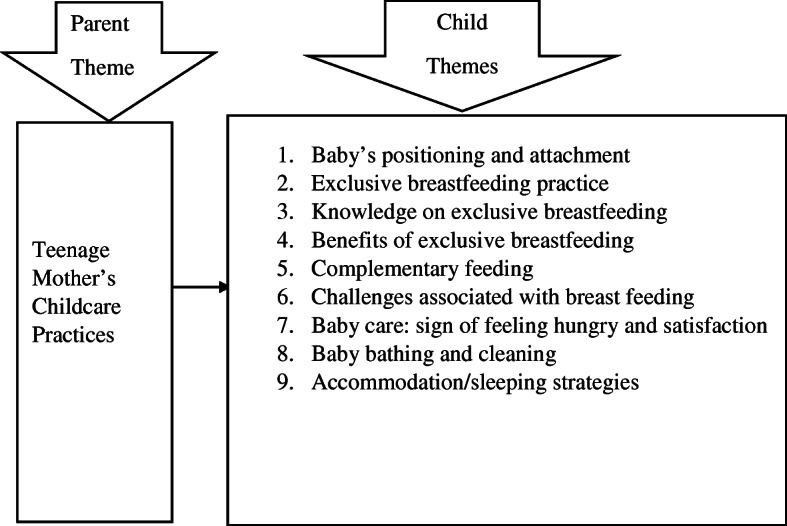
Main theme and sub-themes

Figure 3 presents the main and sub-themes that were obtained from the data analyses. The main theme was the childcare practices of teenage mothers. The sub-themes comprised child positioning and attachment of babies when breastfeeding (exclusive breastfeeding and complementary feeding), child bathing and cleaning practices, accommodation and sleeping arrangements, difficulties and challenges associated with breastfeeding. Most of the pertinent issues gathered from the participants through the in-depth interviews have been described and interpreted.

### Baby’s positioning and attachment practices

Most of the mothers demonstrated healthy and good child positioning and attachment when breastfeeding their babies, irrespective of whether deliveries were at home or hospital. A participant, for instance, had this to say:

*Yes! I sit down and put my baby on my lap and support the head like the one showing in the pictorial guide [picture 2] and I hold the breast in the mouth of my baby. This helps the baby to suck or feed well because if I don’t do it this way the breast milk can pass through the baby’s nose.* ¬R 1, 17 years with a male child.

However, we found that this healthy practice required time for some teenage mothers to master. A teenage mother with a 3 months-old male child who during the first few days following delivery struggled to position her baby when breastfeeding had this to say:

*Yes! But when I started breastfeeding my baby, hmm!, It was very difficult for me because at a point in time I had to use one hand to hold her neck side and use the other hand to control my breast to ensure that I breastfeed the baby well... In my usual ways, I put my baby on my lap and raise his head a bit.* [But now], *I hold my baby the same way it is shown in the book. [Referring to pictorial guide; picture 2]* ¬R 13, 18 years with 3months male child.

### Exclusive breastfeeding practices

Teenage mothers shared their views about exclusive breastfeeding and why they think exclusive breastfeeding is beneficial for every mother to know and practice as a requirement for the child’s growth and development. Some of the study participants indicated that they breastfed their babies well because they know the reasons for and benefits of breastfeeding their babies exclusively. A participant had this to say:

*Yes, I breastfeed my baby all the time, I know the breast milk has all the nutrients my child needs and it is safe and natural. God specifically made it for me to be given to my baby so I have to give the breast milk to my child anytime my child needs it, either in the morning, afternoon, evening and even at dawn to help my baby grow well and get stronger* ¬R 20, 18 years with 3 months female child.

#### Benefits of exclusive breastfeeding

Teenage mothers believed that the only food for their babies in the first 6 months of the baby is breast milk. Almost all participants mentioned at least one benefit or importance of exclusive breastfeeding and accentuated the need to breastfeed. Despite the challenges associated with breast-feeding practices, most of the participants shared the view that it should be encouraged. The following quotes highlight the perspectives shared by the study participants:

*I know that breast milk is the only food for my baby at this time, so I breastfeed her because I know my baby can become strong and healthy when I breastfeed her from now till the time I have to stop breastfeeding.* ¬R 3, 18 years with a female child.

This was corroborated by a 19-year-old mother with a baby aged 4 months old.

*… It helps my baby to grow well and protect my baby against any infections and makes my baby strong and healthy.* ¬R11, 19 years with 4 months child.

### Knowledge of exclusive breastfeeding: the micro-level influence

Teenage mothers are faced with the task of learning to breastfeed their babies through guidance, discipline, nurture, and support. Almost all the teenage mothers mentioned varied means through which they acquired knowledge of breastfeeding. Participants frequently mentioned community health nurses, mothers, friends, family guidance and observation as sources of knowledge on breastfeeding. Teenage mothers were asked about what they know about breastfeeding and sources from which knowledge on breastfeeding was acquired. One participant had this to say:

*My mother supports me and educates me on the kind of food items which, when eaten can increase milk supply* [breast milk] *and shows me how to position the baby for optimal and successful feeding.* ¬R 2, 19 years with 3 weeks child.

Another teenage mother who was practising exclusive breastfeeding based on what the nurse told her when she gave birth at the facility had this to say:

*When I gave birth, the nurse educated me that I should breastfeed my baby for six months before I can give my baby any food so I am just doing that*. ¬R 27, 18 years with a female child.

### Complementary feeding practices

Teenage mothers mentioned why they resorted to complementary feeding of their babies and how those foods or infant formulas also help their babies. Participants whose babies were above 6 months mentioned the various kinds of food they give to their babies and what they think complementary feeding does for their babies. With the aid of illustration in the pictorial guide, teenage mothers mentioned what they feed their babies with as complimentary food, and demonstrated how they do it. Some of the participants had this to say:

*Oooh yes, sometimes I give my baby Cerelac* [a canned baby food] *and porridge [koko] but I prepare the ‘Koko’ at home; I don’t buy it outside … Yes, I use the same position as in the book* [pointing at picture #3 in the pictorial guide] *when feeding my baby. I know when I give other food to my baby, it also makes my baby healthy and stronger.* ¬R 9, 19 years with 7months child.

*… I give my baby porridge, cowbell mix and water for now because the breast milk is not sufficient for her so I have even decided that when she is one-year-old I will stop breastfeeding and give her other heavy food like mashed kenkey, rice, [...] Yes, I put my baby on my lap like it is shown in the pictorial guide number three when I am feeding her.* ¬R 17, 19 years, 10 months female child.

### Challenges associated with breastfeeding practices

Generally, teenage mothers shared the view that there are enormous benefits associated with breastfeeding and that mothers need to breastfeed their babies well. While some reported positive experiences (problem-free), few viewed their experiences to be challenging. Many recalled difficulties that had to be overcome for them to continue breastfeeding for longer. However, the situation was unpleasant for them to continue doing so when there were difficulties with latch, sore nipples, tickling, abdomen and breast pain. It was not the case that teenage mothers who experienced these problems were unable to breastfeed exclusively. However, participants reported some difficulties and unpleasant experiences regarding breastfeeding. These were some observations made by the participants:

*I felt some pain from my stomach/abdomen as my baby suckled, and this continued for a while so I went to the hospital and the midwife told me my womb is getting back to its shape and the doctor prescribed medicine for me but it was still unbearable.* ¬R 22, 17 years, with a female child.

This was further corroborated by a teenage mother aged 18 years with a female child

*I nearly stopped because I did not like putting the breast into my baby’s mouth because it tickles and I feel pain and there was a little [insufficient] breast milk too but I don’t have money to buy those ‘s3’ [like] NAN 1 or ‘s3’ [like] NAN 2 [ … ] I don’t even know how to mention it hm!* [She giggled]*.* ¬R3, 18 years with a female child.

A participant whose husband liked to suck the breast had this say:

*Hmm [ … ] initially, the moment I put the breast into my baby’s mouth and if he starts sucking, I begin to feel for sex but now I am used to it. [She giggled and related that … ] my husband likes to suck my breast ‘paaa’ [a lot] ...* ¬R 28, 19 years with 4 months male child.

### Baby care: signs of hunger and satisfaction

Teenage mothers shared their knowledge and experiences on how they usually get to know when their babies are hungry and signals that suggest they are satisfied. Many of the participants shared similar views. Some noted the signs their babies made when hungry:

*… my child sometimes cries hard* ¬R7; 18 years, with 3 months old child.

With regards to the child’s satisfaction, some participants revealed that they usually pick up signals or sound that their babies usually make when they are satisfied with breast milk. The following are some statements made by one of the teenage mothers:

*When my baby is satisfied, she would reject the breast when I give it to her. When I see that I turn her back against my lap and tap her at the back lightly [ … ] this act of tapping the baby’s back gently is called ‘abatan bo’, the baby will belch. That tells she is full and satisfied [ She giggled].* ¬R 5; 18 years with 5months female child.

### Baby bathing practices

Nearly all the participants mentioned that they get initial support and guidance from their mothers/parent, mothers-in-law, grandmothers and other relatives when bathing their babies. It was evident that the micro-level has much influence on teenage mothers when it comes to bathing and taking care of children, especially during the initial stages of birth. One participant had this to say:

*For the first three months, I had to observe my mother doing it because I did not have the courage to it so she asked me to look carefully anytime she was bathing the baby. After sometimes, my mother helped me to do it. But, nowadays I do it by myself. I even bath my baby twice in a day, one in the morning, one in the evening and I do it very well. Yes, I gently place the baby in the rubber bowl like the one in picture 4* [in pictorial guide] ¬R29; 19 years with 15 months child.

It emerged from the data analysis that bathing children come with challenges for several teenage mothers. Teenage mothers acknowledged that bathing children, especially in the early days of the child require a lot of skills which they lacked. Participants conveyed feelings of fear, lacking in confidence and skills, especially when it comes to cleaning some parts of the body. One participant had this to say:

*I think the little problem I faced when bathing my baby is when I want to clean her vagina. It is very difficult for me because I’m not too sure whether her vagina is clean or not* ¬R 7; 18 years with 3 months child.

Concerning child bathing and cleaning practices, teenage mothers were asked to provide a narrative of how they clean their baby’s umbilical cord. Almost all the participants mentioned that their guardian, parents, mother-in-law or traditional birth attendants (TBAs) assist them at the initial stage. Teenage mothers mentioned they have been using substances such as methylated spirit and cotton wool, capsules mixed with powder, palm kernel oil, and shea butter to the cord stump while others put a wet towel on a hot stone to make it warm and then put the warm towel on the umbilical cord. One participant had this to say:

*When I returned from the hospital my mother was the one bathing and cleaning my baby’s umbilical cord for me. She sometimes uses palm kernel oil mixed with some medicine like ash and black colour [herbs]...I don’t know the name and she rubs it around the chord. She said it relieves the pains and help the wound to heal fast. ¬*R12; 18 years with 2 weeks child.

This was further corroborated by another teenage mother

*I did not clean it myself, the woman [TBA] who helped me to give birth did it for my baby. She puts a stone in a fire and after removing it, she put a wet cloth/towel on the hot stone to make bit warm and apply it on the cord/stump.* ¬R 14; 17 years with a female child.

### Sleeping practices and arrangements

Most of the teenage mothers were found to be living in poor households or environment and had poor sleeping arrangements. Participants were asked to describe where they sleep with their babies. They reported different accommodations such as an extended family house, wooden and metal "container" structures, some of them together with their mothers live in a rented single room. Others live in their family apartment, while some live with their boyfriends/baby’s father. Most of the participants mentioned that they slept on a mat, folded cloth/blanket, with only few of them sleeping on beds with mattresses. Very few of the participants indicated that they sleept under mosquito bed nets. Some of the participants had this to say:

*Hmm, that wooden structure* [pointing at it] *I have a mat [pointed at picture 6 in pictorial guide] that I and my baby sleep together on … sometimes, I feel pains when I wake up but I am used to it. Besides, I don’t have any option*. ¬R 10; 19 years with 20 months old child.

To corroborate this statement, another teenage mother mentioned;

*I was living with my friend at “Esuakyire”* [one of the suburbs of Elmina] *but when I gave birth I decided to come home and stay with my mum. We all sleep in a single room. I have a mat [pointed at picture 6 in pictorial guide] that I and my baby sleep together on it. I put rubber under the baby’s cloth to avoid urine penetrating the mat when the baby urinates on it … I will say it is ok for me and my baby, but I wish we sleep on a good bed and under an insecticide-treated bed net*. ¬R 21; 19 years with 22 months child.

Another teenage mother aged 18 with a baby aged 5 months who sleeps in an old metal container and wanted to prevent malaria had this to say:

*Hmm! Where I stay, there are a lot of mosquitoes because of the big gutter there. So, I always put my baby under a mosquito net. I sleep under it too. Oh yes, like picture 7 in the pictorial guide. I know it’s good to sleep under an insecticide-treated bed net.* ¬R 6; 18 years with 5months child.

## Discussion

This is a qualitative study which explores childcare practices among teenage mothers in KEEA, Ghana. The major childcare practices were child positioning and attachment of babies when breastfeeding (exclusive breastfeeding and complementary feeding), knowledge and benefits of breastfeeding, difficulties and challenges associated with breastfeeding, child bathing and cleaning practices, housing and sleeping strategies/practices. The findings of the study are situated within existing empirical studies and the theoretical framework that guided the study.

Starting breastfeeding early, especially within 30 minutes after birth is a good and recommended practice [11]. Breast milk provides all the food and water that a baby needs in the first 6 months of life and this liquid protects the child from diseases [1, 31]. Our study revealed that most of the teenage mothers appeared to know proper positioning and/or attachment of their babies. The teenage mothers have learned to do so from their immediate environment at the microsystem where they live, including experience from parents (mothers), peers, relatives and mothers-in-law [13]. Others who despite being pregnant for the first time, noted that they had already cared for their younger siblings and had some experiences in childcare practices. This is consistent with existing literature where there is documented evidence of the crucial role of the family, especially mothers in providing education on childcare to young mothers [32]. The childcare experiences of the teenage mothers were based on the use of common practices that were part of their family and community culture instead of scientific-based practices given by health professionals when caring for their babies [2, 32]. Moreover, we observed that some teenage mothers used methylated spirit and cotton wool, while others used ‘traditional means’, such as, herbal medicine mixed with powder, palm kernel oil, shea butter, a hot towel that is heated from hot stone on the cord stump as a common way of cleaning and caring for the child’s umbilical cord. These findings are in line with existing literature where the application of harmful traditional substances, such as cow dung to the cord stump in neonatal care was reported [1, 33].

We observed that teenage mothers experienced several challenges when performing some of the childcare practices. For instance, child bathing and umbilical cord cleaning present enormous challenges to teenage mothers. Hence, most of them usually depended on their parents, mother-in-law, guardians, and TBAs for support. This support is found in the exo-system of the theoretical framework where through education, the community health nurses help teenage mothers to cope better in light of the challenges that they encountered in their maternal roles. This finding is consistent with a study conducted by Gee and Rhodes [34] on the adolescent mother’s relationship with their children’s biological fathers, where they found that adolescent mothers struggled to cope with their new maternal roles.

We also found that knowledge, skills and experiences of breastfeeding were very important in ensuring good breastfeeding practices especially with regards to exclusive breastfeeding. Similar results were reported by a previous Ghanaian study amidst fewer mothers lacking knowledge of healthy feeding practices [35]. The finding of the current study corroborates those found in a study on knowledge of breastfeeding practices in Ghana where it was reported that teenage mothers were practising exclusive breastfeeding successfully, irrespective of the associated challenges and other unpleasant situations [3]. This is because teenage mothers know the benefits of breastfeeding their babies which they learnt through the connection and interaction that exist between them and the various systems (macro, exo, micro and chrono) in the ecological systems theory. Here, experiences and historical perspectives (chrono) and ideologies of culture and beliefs (macro) relating to the importance of breastfeeding informed teenage mothers’ childcare practices. Also, knowledge, skills and guidance from the health and community workers (exo) where older girls observe their mothers/relatives when breastfeeding their younger siblings from their immediate home (micro) are linked in a way that offers a holistic childcare experience [13]. Again, this is in line with Smith et al. [36] who posited that sufficient breastfeeding knowledge is a critical element needed to successfully breastfeed.

Findings of the present study regarding complementary feeding revealed that most teenage mothers generally resort to the use of ‘Koko’ (maize porridge) as first complimentary food and other foods which are made from cereals-based flour such as maize, rice, and cassava. This is part of the cultural values and ideologies of the teenage mothers, the family, and the community which Bronfenbrenner observed in the macro system of the EST. However, these foods may not offer any significant nutritional benefits to the baby. This finding corroborates that of Nti and Lartey’s study on young child feeding practices and nutrition status in rural Ghana, which reported a general use of unfortified porridge with low nutrient as a first complementary food [37]. A similar observation was made by Fjeld et al. [38] where the common complementary food that is introduced from 6 months to infants aged 2 years is maize flour porridge often fortified with vitamin A, salt, and pounded groundnut. Meanwhile, complimentary food or weaning food should, under an ideal circumstance, be clean, contain high energy and protein and easy to digest, culturally appropriate, and locally available as concurred by Arora et al., [39].

The results of this study also revealed a lot of challenges associated with breastfeeding. Teen mothers lacked the experience and confidence to control or overcome these challenges. In a study conducted by Ahmed [40], there was evidence of support for mothers of infants immediately after delivery as a way of overcoming breastfeeding problems and enhancing confidence. However, as conceptualised in the ecological systems theory, teenage mothers will need their mothers or guardians from the microsystem and open-minded healthcare providers that are willing to listen to them and help them overcome challenges with breastfeeding [13]. This is evident in a study conducted by Kridli, Ilori, and Verriest [32] that revealed that the family, mothers, mothers-in-law, and sometimes female relatives play a primary role in supporting the teenage mother in taking care of herself and her new-born after birth.

Regarding sleeping practices of teenage mothers in the communities, we found evidence of inappropriate sleeping arrangements among teenage mothers. These findings corroborate those found in a study by Hanna [41] where the author alluded to the fact that the homelessness of teenage mothers is explained by a variety of factors, including teenage pregnancy, pregnant girls escaping abuse and teenage girls leaving home due to conflict with parents, family members or feeling unloved. Homeless teenage girls or mothers are therefore those who spend the night at lorry parks, kiosk or in abandon/uncompleted buildings. The finding from the current study is in resonance with the conceptual framework where teenage mothers often explore the hopes and dreams of receiving support such as income, health and social services, and other material resources from both the exo and microsystems. The literature further suggests that homelessness or having a poor sleeping condition is common among young mothers, especially in resource-limited settings exposing them and their children to health risks [42].

### Limitations and strengths

Handling issues of trustworthiness in qualitative research are very crucial and determines the worth of the study. In this study, the potential threats to trustworthiness were adequately addressed and demonstrated to avoid researcher and participant biases. For instance, after each interview, the content of the interview was summarised to the participants to be sure if their responses have been captured accurately. However, the decision to exclude teenage mothers with a child above 2 years could be seen as a limitation as it may be argued that an important insight from them might have been lost. Nonetheless, attempting to reduce recall biases, the study, therefore, aimed at getting the pertinent information about teenage mother’s childcare practices.

Besides, situating the study within a theoretical framework adds to the strength of the study. Our study contributes to the existing literature by giving credence to the fact that in many resource-limited settings like Ghana, traditional childcare practices with potentially negative health consequences still exist especially among vulnerable groups such as teenage mothers. Also, our study highlighted the multifaceted nature of the problem. As such, interventions that are aimed at addressing the problem of harmful childcare practices in low- and middle-income countries would have to adopt a holistic approach to dealing with the problem.

## Conclusion

There is a wide range of childcare practices by teenage mothers; both positive and negative. Generally, teenage mothers were inexperienced and found it difficult to properly care for their babies, especially during the early stages of childcare. Whereas few teenage mothers received some sort of support and assistance in caring for their children, they are only limited to the microsystem. We recommend the development of comprehensive approaches that encourage scientific knowledge-based childcare practices needed to improve maternal and child health. Health professionals should be mindful of culture-based traditional childcare practices when developing health education protocols for teenage mothers and ensure that the needed support, skills, knowledge and guidance are provided to ensure their well-being and that of their babies. Thus, support from the exo and macrosystems need to be given adequate attention.

## Supplementary Information

**Additional file 1.**

## Data Availability

All relevant data are within the manuscript. The raw individual participant’s interview transcripts are not publicly available due to confidentiality and privacy concerns. However, data can be made available upon request to interested, qualified researchers through the Department of Population and Health, University of Cape Coast. pop.health@ucc.edu.gh

## Abbreviations

WHO: World Health Organisation
UNICEF: United Nations International Children’s Fund
GSS: Ghana Statistical Service
GHS: Ghana Health Service
SDGs: Sustainable Development Goals
UN: United Nations
EST: Ecological Systems Theory
KEEA: Komenda-Edina-Eguafo-Abrem
IDI: In-depth interview
COREQ: Consolidated Criteria for Reporting Qualitative Research
