# Supplementary material for: Childcare practices among teenage mothers in Ghana: a qualitative study using the ecological systems theory
Source: BMC Public Health. 2021 Jan 4;21:16. doi: 10.1186/s12889-020-09889-7 (PMC7783965; doi:10.1186/s12889-020-09889-7)
Supplement: Supplementary file 1 — Additional file 1. [file 12889_2020_9889_MOESM1_ESM.docx]

APPENDIX A

**UNIVE RSITY OF CAPE COAST**

**DEPARTMENT OF POPULATION AND HEALTH**

INTERVIEW GUIDE FOR TEENAGE MOTHERS

This interview guide is designed to elicit information from teenage mothers (13 to 19 years) with a child two years old or less in the KEEA municipality. The information provided will be strictly used for academic purposes. The information provided will be given the confidentiality it deserves. Please kindly tick in here **[ ]** if you agreed to participate in the study. Thank you for agreeing to participate.

**SECTION A**

**SOCIO-DEMOGRAPHIC CHARACTERISTICS**

1. Age (in completed years)

1. 13 [ ] 2. 14 [ ] 3.15 [ ] 4. 16 [ ] 5. 17 [ ]

6. 18 [ ] 7. 19 [ ]

1. Educational level attained?

1. Primary Level [ ] 2. J.H.S [ ] 3. S.H.S [ ] 4. Tertiary [ ] 5. Other, please specify…………………………………

1. Religious affiliation

1. Christian [ ] 2. Islamic [ ] 3. Traditionalist [ ] 4. Other, please specify…………

1. Marital Status

1. Never married/single [ ] 2. Married [ ] 3. Divorced [ ] 4. Separated [ ] 5. Widow [ ] 6. Other, please specify ………………………………

6. Current occupation ……………………………………

**SECTION B**

Thank you for agreeing to speak with me today, as you will remember from the things I told you earlier if you have any question or concerns during our conversation today, please kindly feel free and let me know ok!. [Notes to the interviewer: Probe questions are those bulleted]

**Warm-up questions**

Tell me about your child.

- Which day was your child born, is your child a male or female, how old is your child, is your baby big or small, etc.? *(Ask general questions to ensure participant feel comfortable)*
- What do you like about your baby?

**Objective 1: Assess teenage mother’s child care practices**

We are going to talk about some child care practices

1. Do you breastfeed your child? How do you breastfeed your baby? *What do you see in the picture (show the pictorial guide)?*

- Can you show me how you hold/position your baby when you breastfeed?
- Why do you think it is important to position your baby this way when you breastfeed?
- How do you know if your child is still hungry and/or satisfied after breastfeeding?
- Please describe to me any uncontrollable and/or unpleasant experiences when you breastfeed your child? Probe for views on *how* and *why* this happened!

1. Do you breastfeed your baby exclusively? *Why*?

- What do you know about exclusive breastfeeding?
- Why do you think it is beneficial to your child?

1. Do you give your baby complementary food? ***Why*?** *(Encourage the participant to provide detail information on the timing and other issues relating to complementary feeding).*

- What do you think about complementary feeding? *What do you see in the picture* *(Show the pictorial guide)*
- Can you show me how you give complementary feeding?
- Why do you think providing other liquid or formulas does for your baby?
- Why do you give your baby formulas apart from breast milk? Tell me more.

1. What are the challenges you face when breastfeeding your child? ***Probe but don’t prompt!*** E.g. *nipple sore, tickling, insufficient milk etc.?*

- How do you address this problem(s) you have mentioned? *Ask this in relation to the problem mentioned*

1. Do you usually bath your child? *What do you see in the picture (show the pictorial guide)*
2. Can you show me how you hold or position your baby when bathing him or her?
3. Which of the child bathing position *(in the pictorial guide)* do you think is the best (safe) practice? Why?

- What are the things you use to bath your child and how many times do you bath your child in a day? *Why*?
- What are the challenges you face when bathing your child?
- How did you address that challenge? *Ask this in relation to the challenge mentioned*
- How did you care for your child’s umbilical cord? For example, Things you use, how you use it?
- Who else helps you to clean the baby? *interview the person for views on how and whether the things they use is clean or appropriate*

1. Please can you describe where you and your baby sleep?

- *What do you see in the picture (show the sleeping arrangements in the pictorial guide)*
- Which sleeping arrangement do you have/use?
- Why do you think (this one) is appropriate than the others?
- *Ask this in relation to the particular sleeping arrangement preferred/chosen*

1. Apart from you who else takes care of your baby? (*interview the person shortly if available and willing to talk*)

- Please can you tell me about the things you do to support the teenage mother and why? *(Probe for details)*

1. Is there anything that you would want to say about the things we have discussed today?

**Thank you for your time and participation. I wish you well and have a nice day**

APPENDIX B

PICTORIAL DIARY INTERVIEW GUIDE

PICTORIAL DIARY


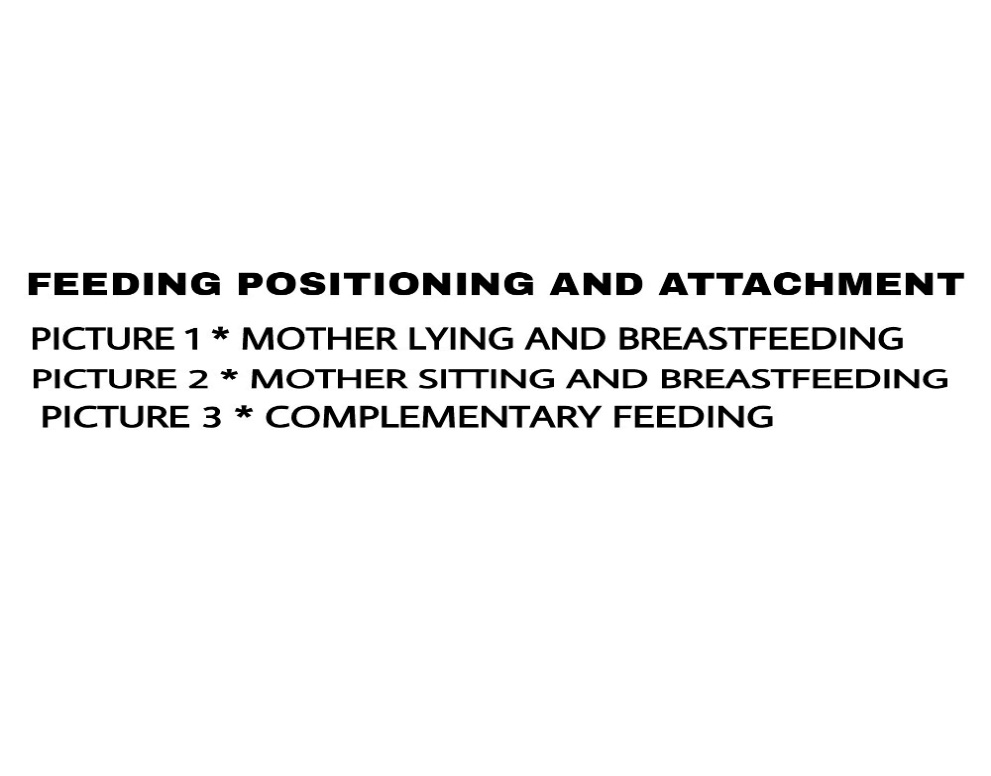
**PICTORIAL GUIDE FOR TEENAGE MOTHERS’ EXPERIENCES WITH CHILDBIRTH**

**
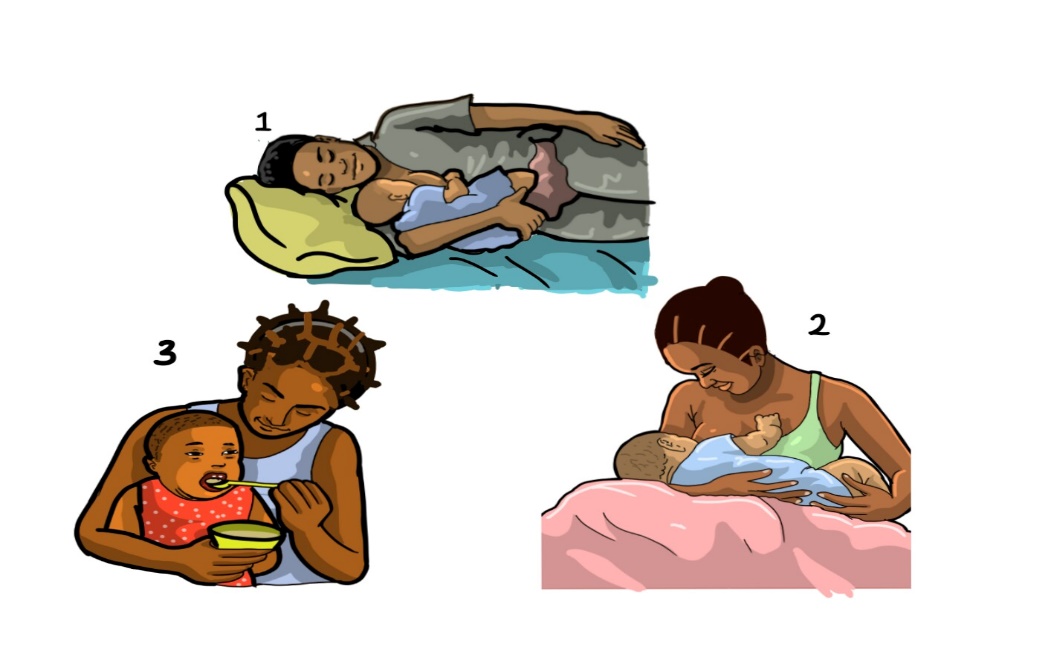
**


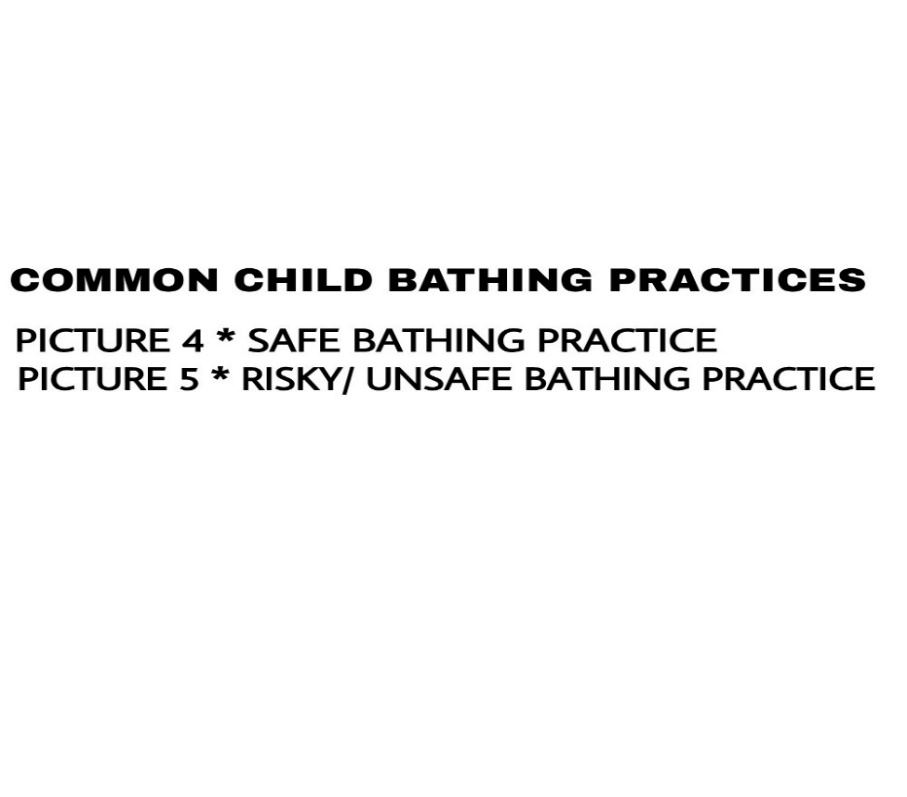

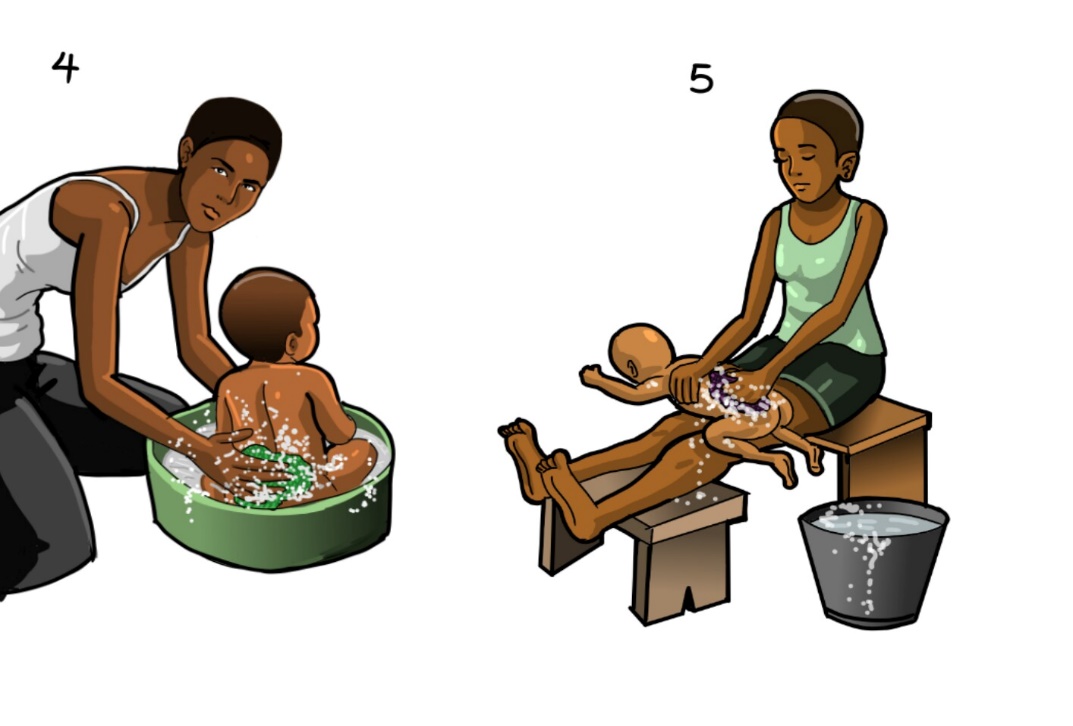


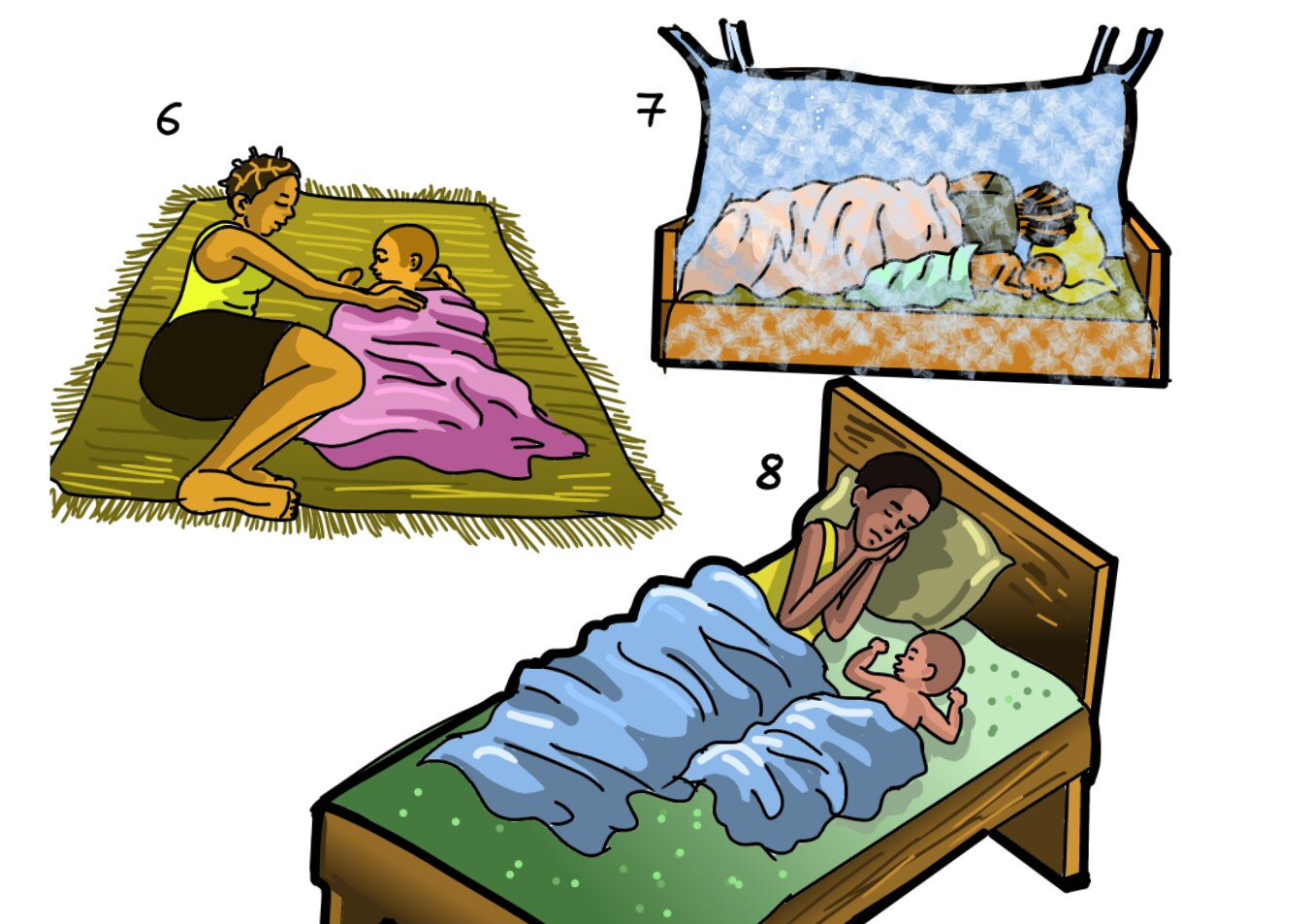

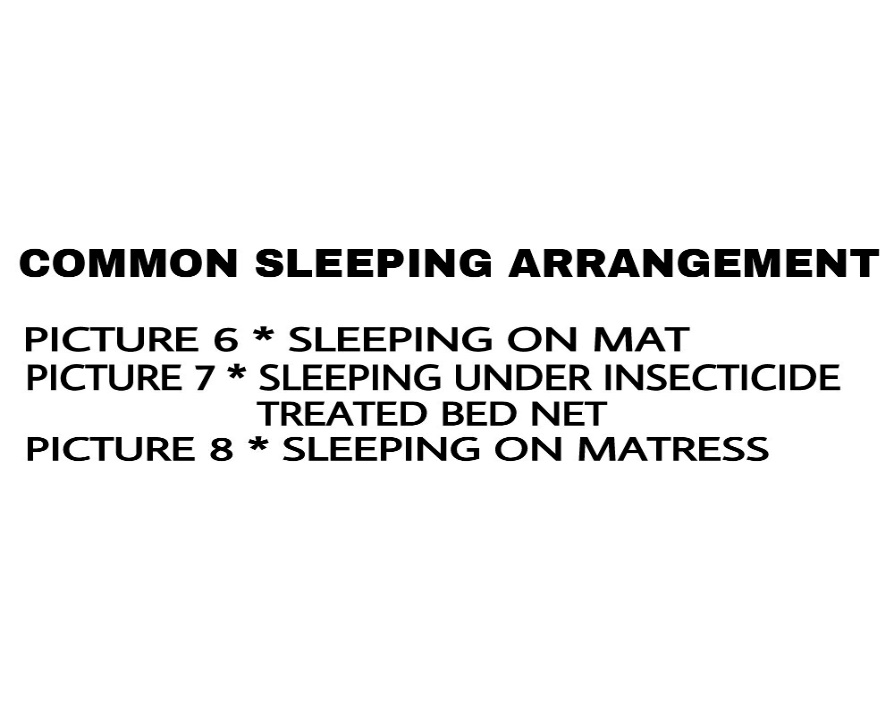


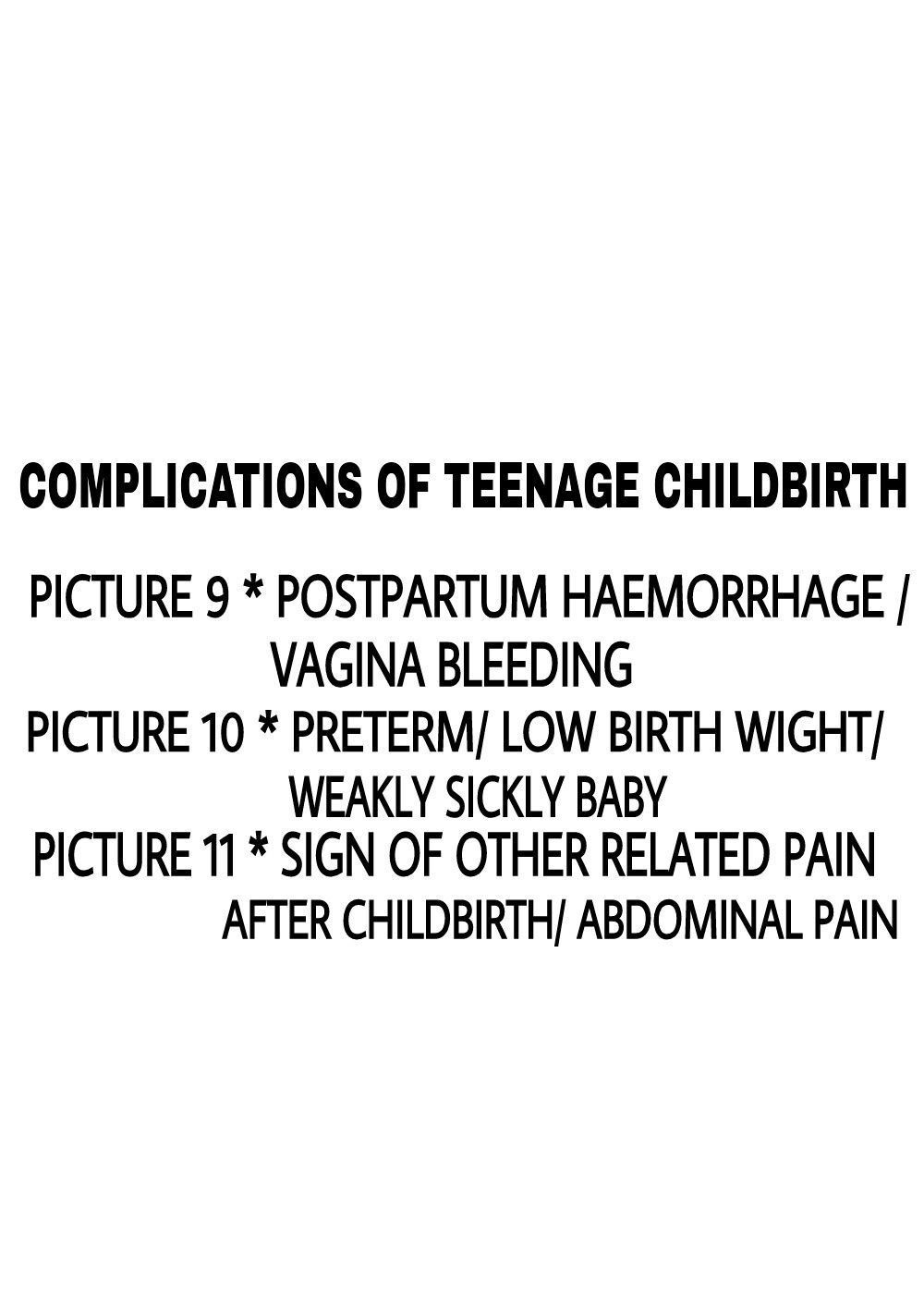

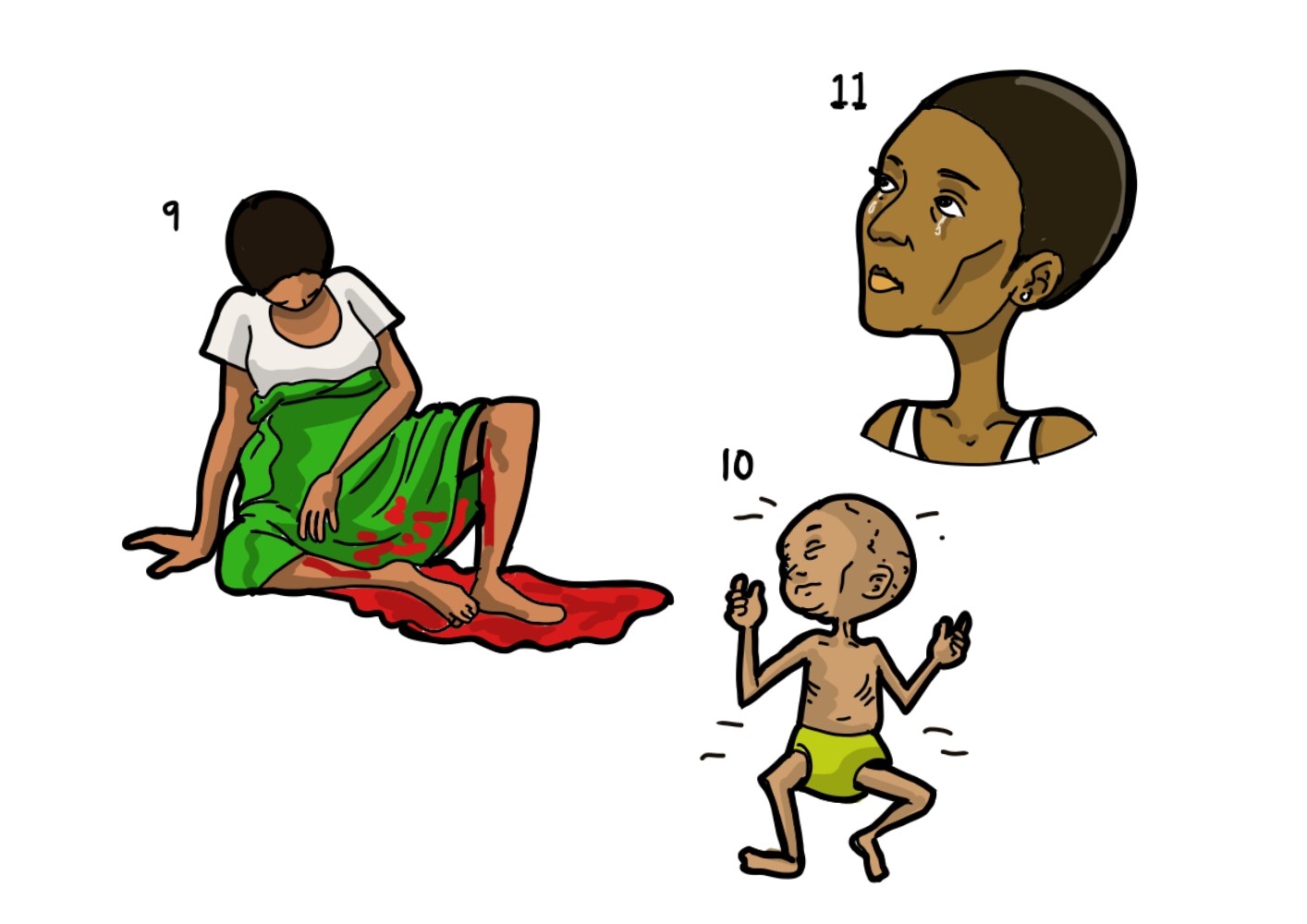


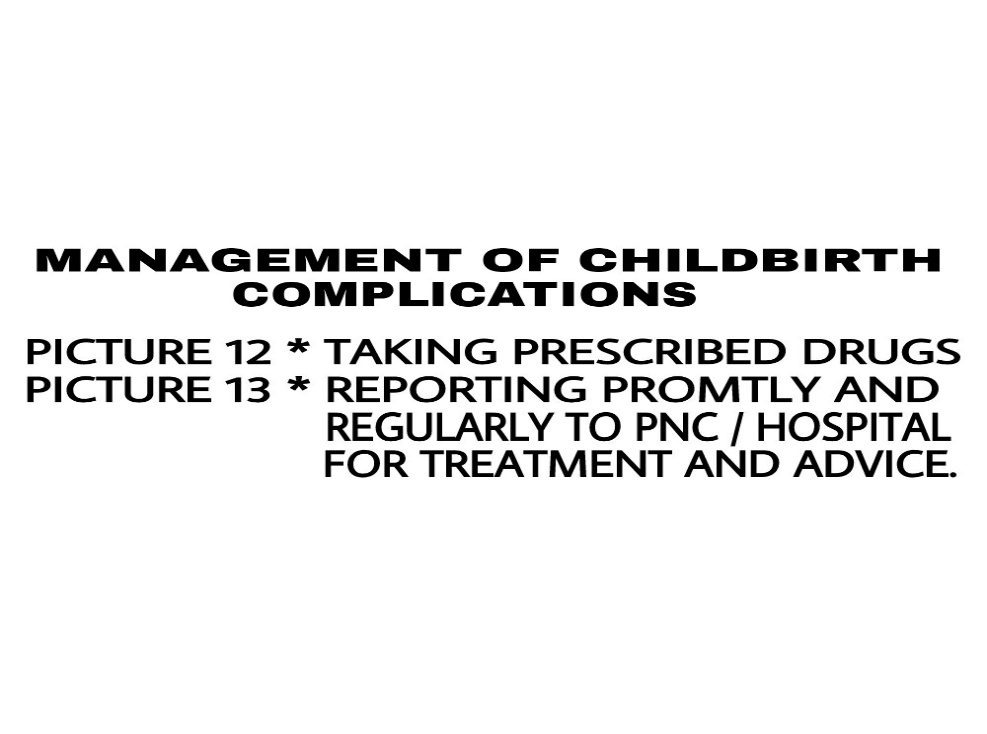

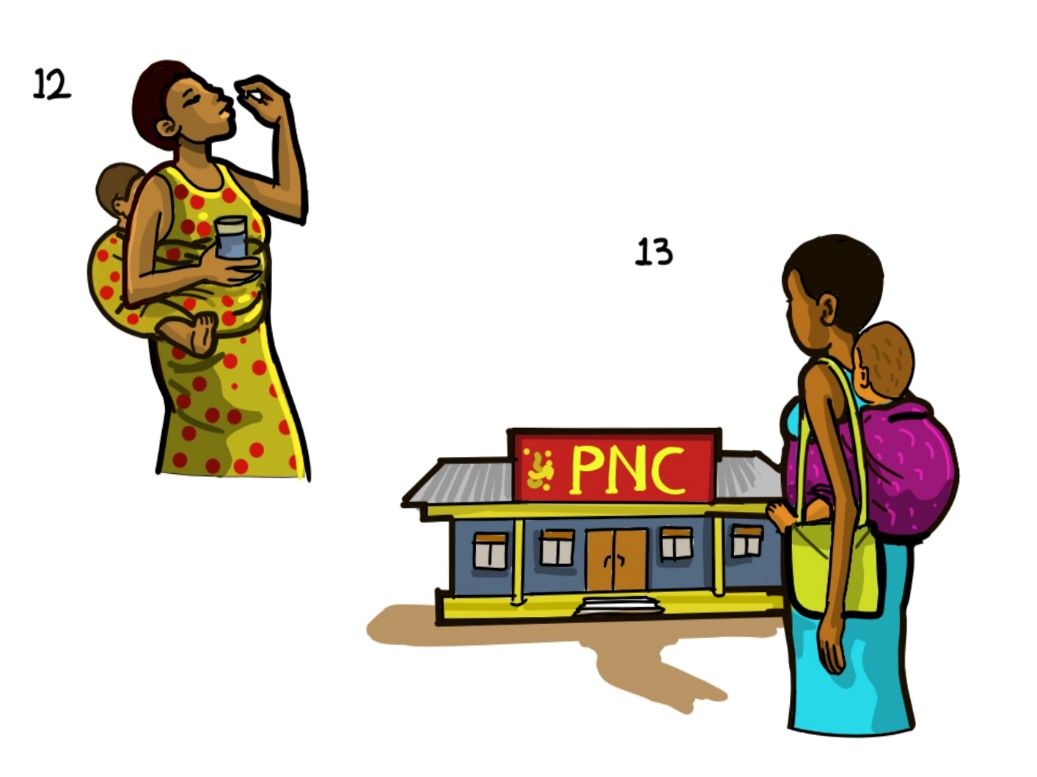


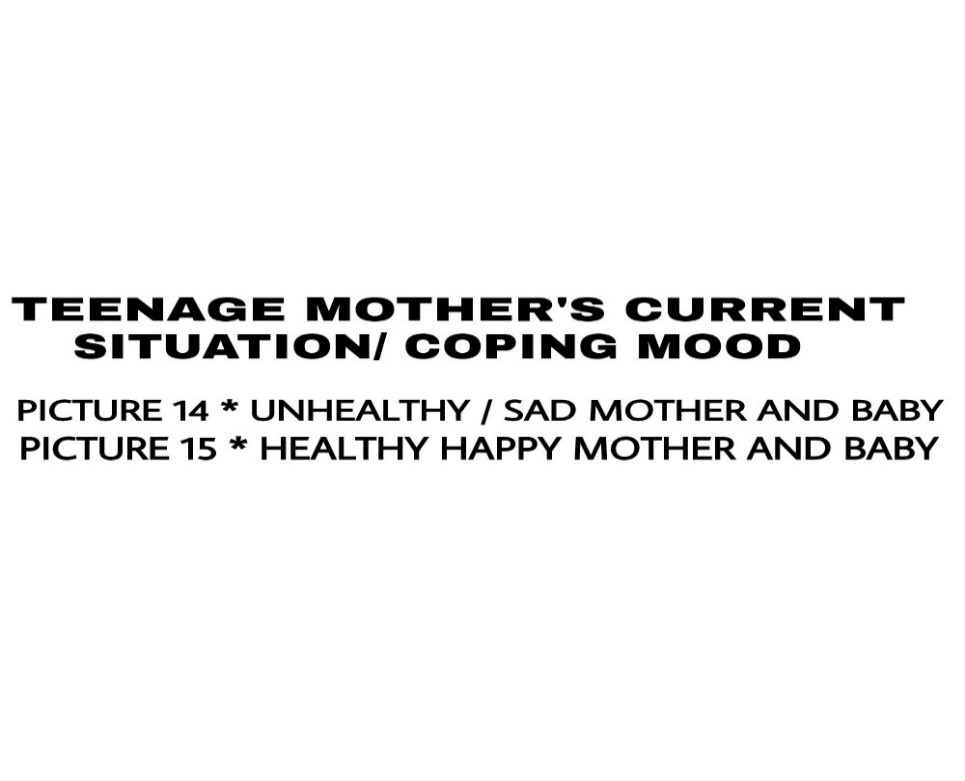

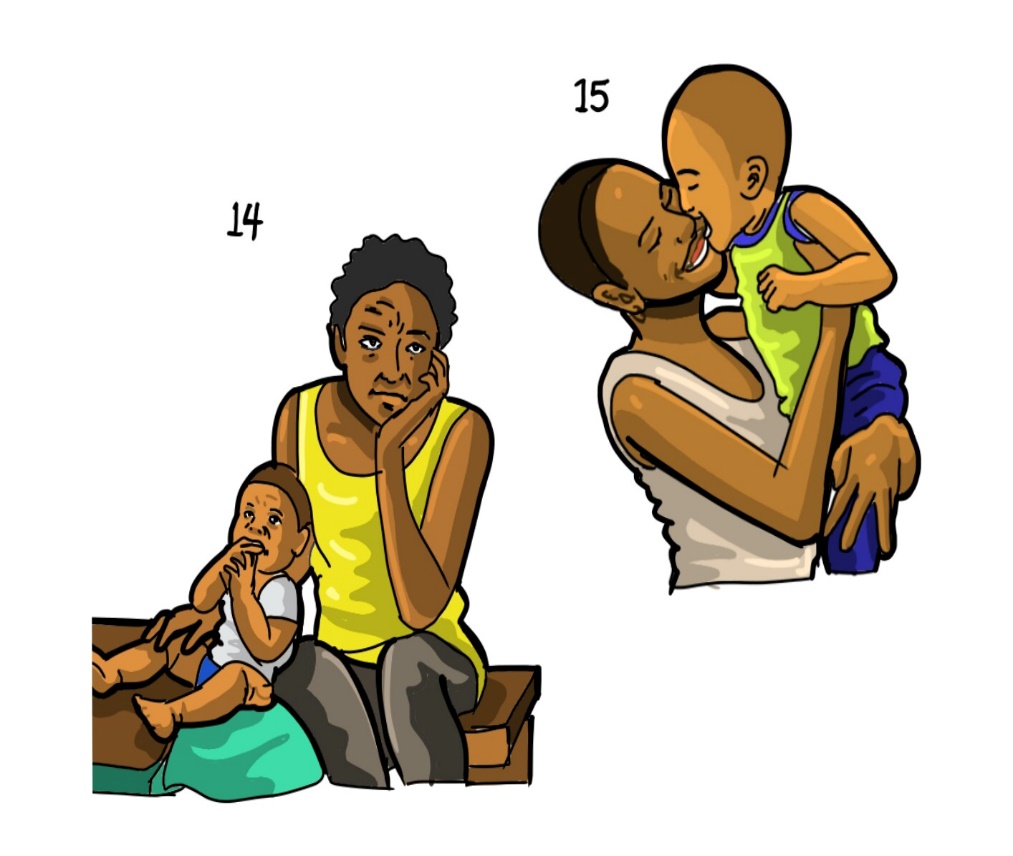


**THANK YOU FOR YOUR TIME AND PARTICIPATION.**

**I WISH YOU WELL**
